# VATS bullectomy and apical pleurectomy for spontaneous pneumothorax in a young patient with Swyer-James-Mc Leod syndrome: case report presentation and literature review focusing on surgically treated cases

**DOI:** 10.1186/1749-8090-9-13

**Published:** 2014-01-10

**Authors:** Nikolaos Panagopoulos, Gerasimos Papavasileiou, Efstratios Koletsis, Myrto Kastanaki, Nikolaos Anastasiou

**Affiliations:** 1 Department of Thoracic Surgery, “Olympion” General Clinic of Patras, Patras, Greece; 2 Department of Thoracic Surgery, “Agii Anargiri” General Oncological Hospital of Kifissia, Athens, Greece; 3 Department of Cardiothoracic Surgery, School of Medicine, University of Patras, 31 Chlois Str, 16673 Voula, Athens, Greece

## Abstract

**Background:**

Swyer-James-McLeod Syndrome (SJMS) is an uncommon, emphysematous disease characterized by radiologic hyperlucency of pulmonary parenchyma due to loss of the pulmonary vascular structure and to alveolar overdistension.

**Case report:**

We herein describe a 15-year-old Caucasian patient with well-established SJMS since childhood who presented with spontaneous pneumothorax. Video-assisted thoracoscopic bullectomy with apical pleurectomy was performed. Since SJMS is considered an on-going inflammatory process, the patient one year after surgery exhibits excellent quality of life with no pneumothorax recurrence.

## Background

Unilateral pulmonary emphysema or unilateral hyperlucent lung with secondary deficiency of blood supply was first described by Swyer and James [1] in a six year old child. Because of recurrent infections right pneumonectomy was performed and the underlined emphysematous condition was considered acquired in origin. One year later McLeod published his work [2] referring to clinical, presented the radiological and bronchographic characteristics of nine patients with one lung transradiency and decreased breath sounds. There was neither obstruction of the main bronchi nor gross bullous emphysema. The disease presented a distinctive pattern but the underlined pathology was unknown. Today, Swyer-James-McLeod syndrome (SJMS) is a well-established clinico-pathologic entity characterized by obliteration of the small bronchioles, hypoplasia/or absence of pulmonary artery and peripheral vascular bed and emphysema [3,4]. The syndrome is thought to be secondary to bronchiolitis obliterans acquired in infancy or childhood resulting in obstruction of small airways and concomitant emphysema. Hypoplasia of lung vessels is also considered to be secondary to chronic inflammation [5].

Patients suffering from SJMS may be asymptomatic for many years or suffer from recurrent episodes of pulmonary infections. They usually present with chronic productive cough and dyspnea upon exercise. Rarely do they present with symptoms of spontaneous pneumothorax due to rupture of an emphysematous bulla secondary to the inflammatory process.

Generally, the majority of patients with SJMS were treated conservatively. Surgical therapy was rarely performed for symptomatic patients mainly adults and most of them by open thoracotomy procedures of major lung resection such as pneumonectomies and lobectomies.

Spontaneous pneumothorax is considered an emergency condition particularly among patients with SJMS due to the underlying pathologic disease. We present a case of a 15-year old patient with SJMS presented with spontaneous pneumothorax due to emphysematous bulla rupture. The patient underwent video-assisted thoracoscopic (VAT) bullectomy with apical pleurectomy and reinforcement of the staple line with bovine pericardium in order to prevent any recurrence of the pneumothorax in the future and minimize postoperative air leak. Additionally we make a brief review of the literature focusing in those reports adopting the surgical approach as a route for therapy.

## Case report

Our patient is a 15-year old Caucasian basketball player with a well-established diagnosis of SJMS. In the age of five years he had a serious pulmonary infection with Mycoplasma Pneumoniae for which he had to hospitalize. After his exit from the hospital and up to the age of ten years he had repeated episodes of pulmonary infections with bronchitis and dyspnea. It was that time were the diagnosis SJMS was made due to bronchiolitis obliterans and resulting emphysema. The radiologic exams were unremarkable. Chest X- ray showed a hyperlucency on the left pulmonary field with the left lung being relatively smaller than the right. Upon lung function tests the child exhibited measurements of lung obstructive disorder with decreased forced expiratory volume in 1 sec (FEV1) and forced vital capacity (FVC) in relation to the normal value considering his age and somatometric parameters. Moreover, the ratio of (FEV1) to FVC (FEV1/FVC) was decreased, indicating airway obstruction. Pulmonary scintigraphy with ^99m^Tc showed decreased perfusion of the affected lung mainly at the apex, whereas the right lung remained unaffected. The patient continued to exhibit recurrent episodes of bronchial infections up to the age of ten. Since then and for the next five years he remained asymptomatic.

**Figure 1:**
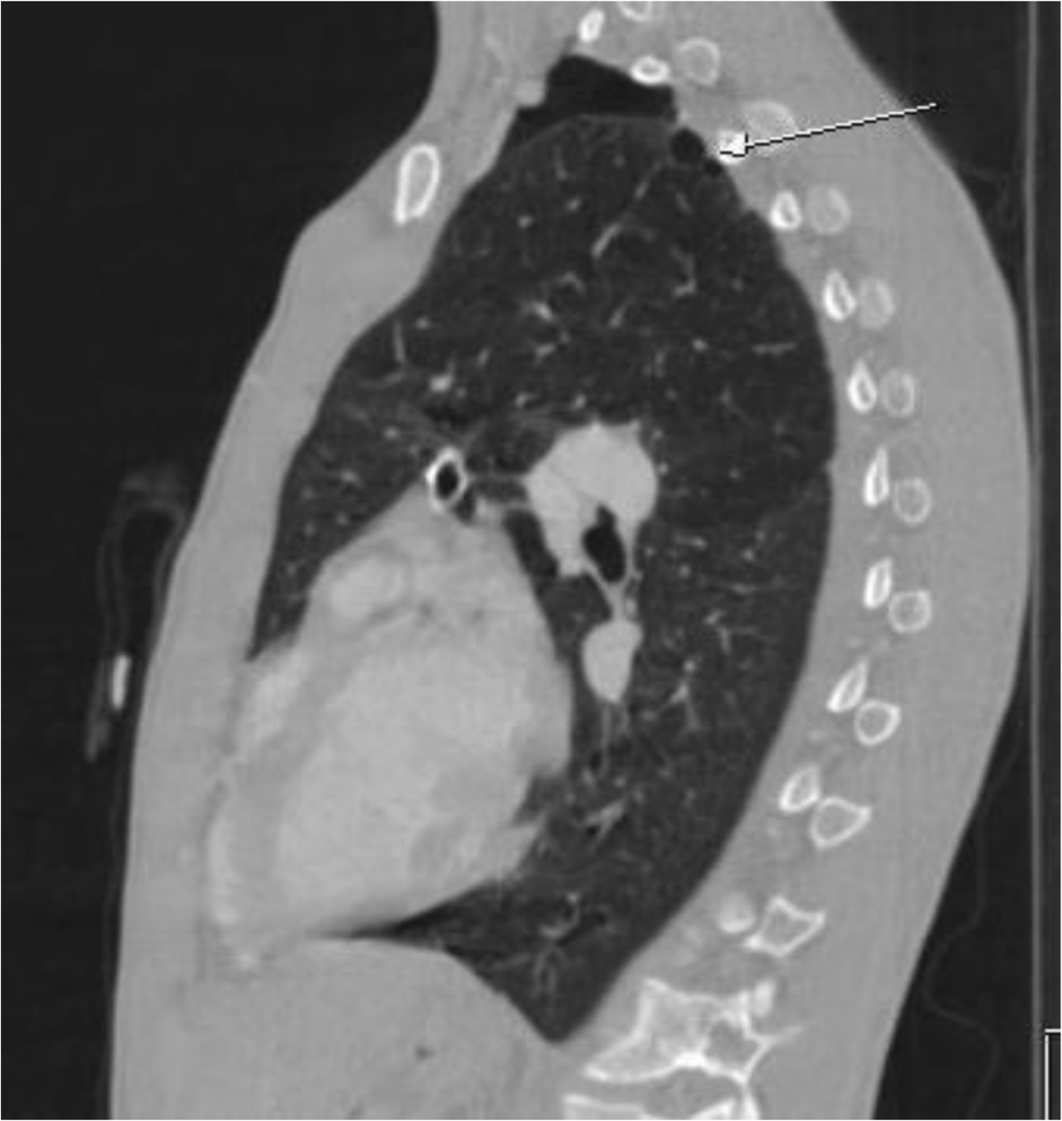
**CT scan angiography in our patient with SJMS, showing remaining pneumothorax with presence of apical emphysematous bulla in the left lung (arrow).** Note hyperlucency of the upper pulmonary lobe in comparison to the lower.

**Figure 2:**
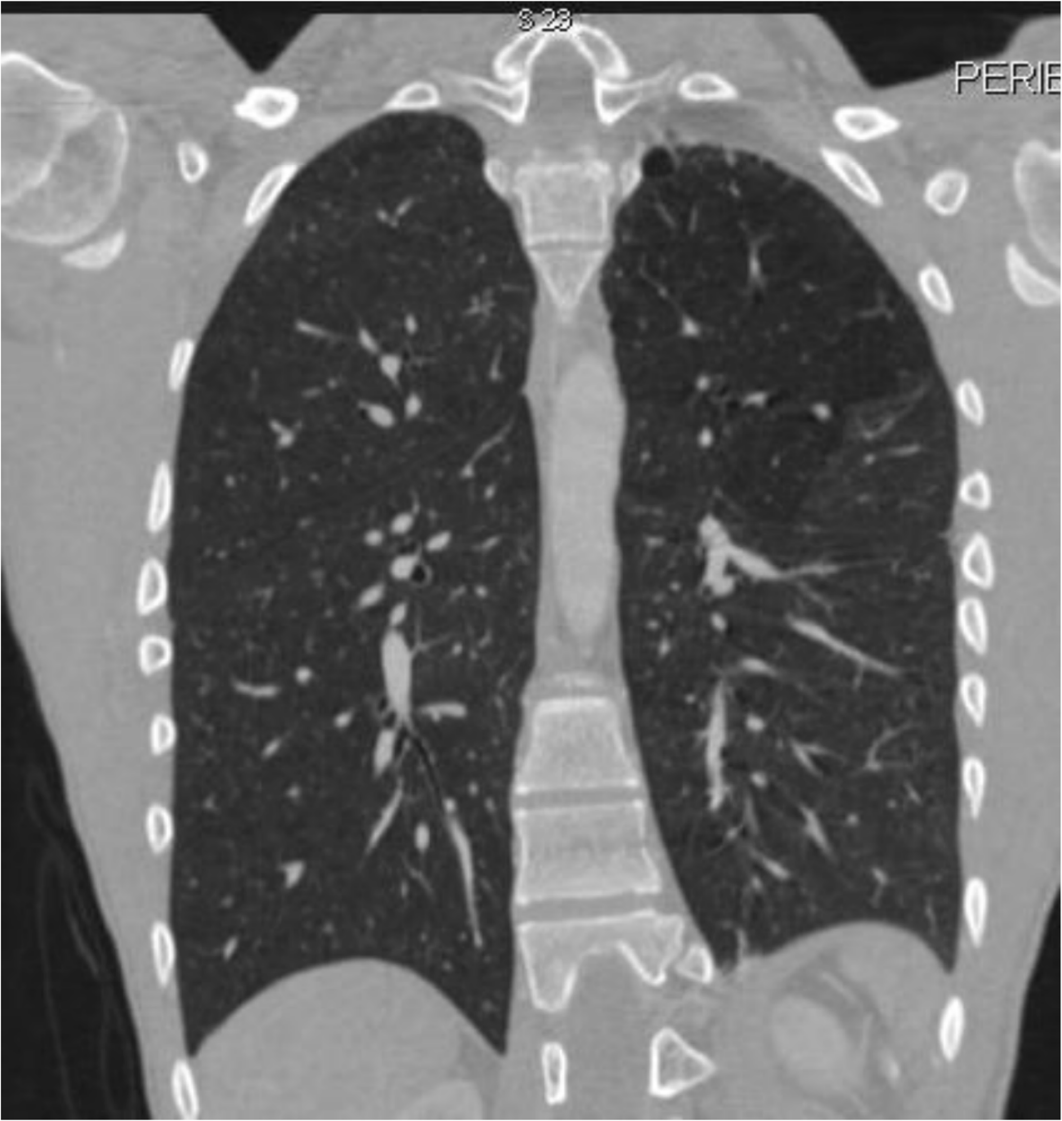
**CT scan angiography of the same patient.** Note the decreased vascularity of the left lung with hyperlucent upper lobe and presence of apical bulla. The left lung is smaller in comparison to the right.

One year ago the patient was admitted in the emergency department complaining of sudden onset of dyspnea accompanied by pleuritic chest pain on the left side. He has been feeling well over the past days, was afebrile and reported no history of recent trauma. Auscultation revealed decreased breath sounds on the left. A chest X-ray followed revealing spontaneous pneumothorax on the left side. Immediate insertion of an 18 Fr thoracic chest tube in an apical position for the evacuation of air and the patient was transferred to the ward for further hospitalization. During the next few days the patient continued to exhibit significant amount of air in the underwater seal mainly during coughing and the chest X-rays showed that the pneumothorax still existed; although significantly decreased in size. Chest CT angiography followed showing an emphysematous bulla 3 cm in diameter in the left upper lobe with partially expanded lung and small remaining pneumothorax. Additionally, hyperlucency of the left upper lobe with decreased vascularity in concordance with SJMS were also present (Figures 1 and 2). Patient’s underwater seal drain was changed and connected to negative suction in order to remove the remaining air and expand the lung. He remained on negative suction for the following seven days with no radiological or clinical improvement; therefore, surgical therapy was decided.

Under general anaesthesia using double lumen endotracheal tube, VAT- bullectomy of the left upper lobe was carried out through 3 access ports that were 5.5, 5.5- and 20-mm in diameter. Bovine pericardial sleeve used to buttress and reinforce the staple line. Apical pleurectomy just over 5 cm followed to achieve mechanical pleurodesis.

Postoperatively patient’s underwater seal continued to remain connected to mild negative suction (10-15 cmH_2_O) for five days in order to accomplish full pulmonary expansion since he was emphysematous with associated obstructive bronchiolitis and decreased pulmonary elasticity. No postoperative air leak was observed. During his stay we observed minimal apical space when the patient was off suction. Therefore we decided to connect the intercostal drain to a one flutter valve bag (Portex bag) and we discharge him home to optimize his mobilization and treat the minimal apical space. The chest drain was on the 15^th^ postoperative day. Chest X ray after removal showed fully expanded pulmonary parenchyma. One year postoperatively he is in excellent clinical condition and able to cope fully with his basketball activities. No recurrence of spontaneous pneumothorax is observed.

## Discussion

SJMS is an uncommon disorder. Among 52 patients with bronchiolitis obliterans, Gosink et al. [6] reported only two cases of hyperinflation. The diagnosis is usually based on radiologic and clinical findings rather than on the results of pathologic examination. Fregonese et al. [7] reported prevalence of 0.01% in a survey of 17,450 chest radiographs.

SJMS is characterized by the presence of constrictive bronchiolitis with dilatation and destruction of alveolar structures, resulting in significant air trapping and lung. Although classically involving an entire lung, the disorder can be lobar or segmental. The main pathogenetic event seems to be acute bronchiolitis with obliteration of small airway in infancy or early childhood. The disease starts as an obliterative bronchiolitis with concomitant vasculitis commonly following infections with certain organisms. Consequent damage to the terminal or respiratory bronchioles in early childhood prevents normal development of their alveolar buds [8].

Bernardi and associates [9] studied 2 patients with the syndrome examining the cytological and immunophenotypical profile of bronchoalveolar lavage (BAL) and suggested an ongoing active process in the lung with inflammatory characteristics. Thus the condition should be differentiated from congenital anomalies of airway/pulmonary vessels and bronchial obstruction due to mucus plug or foreign body [8].

Respiratory infections associated with bronchiolitis obliterans and the manifestation of SJMS include measles, whooping cough by Bordetella pertussis, tuberculosis, Mycoplasma pneumonia, influenza A. and adenoviral infections with the subtypes 3, 7, and 21 [10]. Additionally, bronchiolitis obliterans has been associated with other non-infective causes, including aspiration, toxic fumes, and organ transplantation [11].

The onset of symptoms typically occurs during infancy or early childhood in association with frequent respiratory infections. Patients present usually with productive cough, shortness of breath, and dyspnoea on exertion. Hemoptysis may be seen occasionally. These symptoms correlate with the degree of bronchiectasis present. Patients with little or no bronchiectasis have minor symptoms or are asymptomatic and may remain undiagnosed until adulthood. Moreover, adult patients with SJMS are often diagnosed after a chest radiograph obtained for another reason [12]. As already mentioned, severity of symptoms is influenced mainly by the presence or absence of saccular bronchiectasis. Patients without or with cylindrical bronchiectasis may present with mild respiratory symptoms and a spontaneous tendency to improve, those with saccular bronchiectasis have repeated episodes of pneumonia, and their clinical features and evolution resemble those of patients with classic postviral bronchiectasis [12,13].

The diagnosis of SJMS requires the exclusion of other causes of unilateral hypertranslucency, since this particular finding correlates with several diseases of either the lung parenchyma or vasculature. Parenchymal diseases include congenital lobar emphysema, bronchogenic cyst, pneumatocele, bronchiectasis with air trapping and emphysema secondary to bronchial stenosis or bronchospasm. Congenital vascular disorders such as pulmonary artery agenesis or hypoplasia or acquired disorders that create stenosis or compression of the main pulmonary vessels may cause radiographic hyperlucent lung images [7,14].

Bronchiolitis obliterans results in inflammation and fibrosis, causing narrowing of respiratory bronchioles lumens [15]. Fibrosis of the interalveolar septae causes obliteration of the pulmonary capillary bed and secondarily diminishes blood flow to the major pulmonary artery segment. This reduction in flow creates hypoplasia of the pulmonary arterial bed. Moreover, the reduction in ventilation causes a compensatory decrease in perfusion [12]. Hyper-expansion of the terminal air sacs secondary to bronchiolar obstruction of the peripheral airways offers additional mechanical resistance to flow through the alveolar capillaries and contributes to atrophy of the vascular beds [16].

Despite characteristic findings by chest radiography, scanning with CT is the imaging technique of choice in establishing diagnosis of SJMS [13]. Although one lung or one lobe is usually affected, the contribution of CT scan clarified that the disease may be more heterogeneous in distribution and contralateral parenchymal lesion may also be present [17]. Additionally, the volume and antero-posterior attenuation gradient of the affected lung, size and distribution of central and peripheral pulmonary arteries, the degree of air trapping, the patency of main airways and presence of bronchiectasis are investigated. Also, by excluding central obstruction CT scan make bronchoscopy unnecessary [18].

Ventilation/perfusion (V/Q) scan is very helpful in determining the extent of the disease and correlates well with high resolution computed tomography (HCRT) which seems to be the most appropriate technique. V/Q scans document matched ventilation and perfusion defects [19].

In the majority of cases suffering from SJMS, therapies are primarily conservative and supportive. Antibiotic therapy, use of bronchodilators and chest physiotherapy with postural drainage of the secretions may be helpful. In rare cases, however, patients with SJMS are treated by lung resection for intractable disease. Surgery should be reserved as the final solution for therapy and indicated when all other means of therapy are ineffective, especially when treating younger patients since pneumonectomy at this age is considered an amputating procedure. According to symptoms, patients with SJMS can be classified into the following categories regarding surgical indications: patients presenting with recurrent infections not improving with conservative support [13,20,21]; patients with clinical deterioration and resulting failure to thrive [7,14,22-26]; and finally those presenting with spontaneous or recurrent pneumothorax [27,28]. Kim et al. [29] reported surgical therapy in a 5 year old patient due to pulmonary hypertension.

Most patients treated surgically for SJMS are adults (20–68 years old). Only in 3 published studies patients were of younger age (11, 15 and 5 years old) [7,28,29].

The majority of studies adopting the surgical approach for SJMS describe open thoracotomies with major pulmonary excision. Pneumonectomies are described in 5 studies [7,14,20-22], lobectomies in 3 studies [13,23,27], whereas segmentectomy is performed in one case [24]. Alternatively, Vishnevsky et al. [25] reported occlusion of the main bronchus as an option instead for pneumonectomy in adults with SJMS. They supported that the occluded lung acts as a biologic prosthesis and minimizes mediastinal displacement and subsequent hyperexpansion of the healthy lung. Bronchial occlusion was carried out through a small thoracotomy contributing to a lesser traumatic area. However, this surgical approach by means of occlusion of the main bronchus leaving the affected lung in place should be reserved when there is no blood flow to the affected lung since it may lead to a marked ventilation/perfusion mismatch and subsequent hypoxemia.

Taking into account the advantages of minimal invasive techniques in thoracic surgery such as decreased postoperative pain, decreased need for postoperative analgesia, improved postoperative pulmonary function, shorter hospital stay and better cosmetic results, it would be reasonable to consider those Video-Assisted Thoracic Surgery (VATS) techniques as the appropriate therapeutic approach instead of thoracotomy, for patients presenting with debilitating symptoms of SJMS; especially when treating younger individuals.

To the best of our knowledge, two studies have adopted minimal invasive approaches for surgery in SJMS. In the study of Tasaki and associates an adult professional bicycle racer with SJMS exhibiting persistent respiratory distress upon exertion had been submitted to lung volume reduction surgery (LVRS) with video-assisted thoracoscopy (VAT). The patient postoperatively showed progressive improvement in respiratory function and became completely asymptomatic during a bicycle race. In another study of Inoue and associates, a 15-year old SJMS patient with spontaneous pneumothorax underwent VAT bullectomy. To accomplish pleurodesis, the authors used minocycline as a chemical agent and did not perform mechanical pleurodesis. The patient ten months after surgery showed recurrence of the pneumothorax and had to be reoperated through VATS. Intraoperatively they observed formation of new bullae in the lung parenchyma. Pleural abrasion in addition to chemical pleurodesis again with minocycline was performed during the reoperation. Additional chemical pleurodesis before extubation in the ward was carried out. Eighteen months after reoperation there was no pneumothorax recurrence.

Our patient a 15 years old male, basketball player, with well-established diagnosis of SJMS since childhood, presented in the emergency department with signs and symptoms of spontaneous pneumothorax confirmed by clinical and radiologic examination. A thoracic chest tube was inserted to evacuate the air from the thorax but the patient exhibited no major clinical or radiologic improvement, with continuous air leak from the drain even after the application of negative suction on the underwater seal. Video-assisted thoracoscopic bullectomy was combined with mechanical pleurodesis by performing apical pleurectomy of the parietal pleura. No addition of any chemical agent was used.

Recurrence of pneumothorax after VATS procedures have been described in many reports, with recurrence rates ranging from 5% to 15% [30,31]. Moreover the recurrence of pneumothorax after VATS bullectomy procedure is significantly increased in contrast to bullectomy via thoracotomy [32]. Studies suggest that the long term outcome of patients undergoing VATS stapled bullectomy as a radical therapy for spontaneous pneumothorax was unsatisfactory. A symphysial procedure should therefore be added to VATS stapled bullectomy in order to prevent long-term postoperative recurrence. Moreover, they observed that the addition of pleurodesis to VATS bullectomy had no disadvantages versus bullectomy alone in worsening postoperative chest pain or pulmonary function [30].

Those patients who were reoperated for recurrent pneumothorax the addition of pleurodesis during the first surgery didn’t have any serious impact upon dissecting the adhesions during reoperation. Surgeons were able to dissect the adhesions easily since they appear loose and scattered [30]. On the other hand it is well known that if the primary procedure is an open thoracotomy then reoperation for any reason is vigorous due to dense and diffuse adhesions created by a greater traumatic surface.

Focusing on pleurodesis it can be of two types: mechanical or chemical. Mechanical pleurodesis can be performed either by: (a) apical pleurectomy by removing a substantial area of the parietal pleura from the apical thoracic cage or, (b) abrasion of parietal pleura. Our preference is to perform apical pleurectomy in relation to pleural abrasion since it has been shown that the latter technique did not decrease the recurrence of pneumothorax after VAT wedge resection of bullae for spontaneous pneumothorax. Younger individuals were associated with higher risk of recurrence [33].

Chemical pleurodesis by adhesion formation can be accomplished by installation of various substances such as talc [34], antibiotics [35], glucose solution [36], blood [37], in association with or without pulmonary excision. Combination of mechanical and chemical pleurodesis has also been used with various results [36,38]. Results supporting one method over the other (mechanical versus chemical) are inconclusive [39].

Bovine pericardial patch as sleeve in order to buttress the stapling line has been used for more than a decade during surgical procedures for emphysema. Results show patients in whom pericardial patch buttressing was used in the staple line showed earlier removal of the chest tubes due to minimal postoperative leak, earlier hospital discharge and cost effectiveness due to shorter time of stay in comparison to those which pericardial patch was not used, as reinforcement [40].

Spontaneous pneumothorax is an emergency situation which sometimes necessitates thoracic surgery. Among patients with SJMS and because of their underlying pathology, spontaneous pneumothorax can be a troublesome condition. In this case we prefer to perform VAT bullectomy and buttressing of the staple line with bovine pericardium with apical pleurectomy for mechanical symphysis. The result is twofold: apical pleurectomy is an excellent symphysial procedure which, with the addition of buttressing of the staple line with bovine pericardium, prevents pulmonary collapse and diminishes prolonged postoperative air leak, minimizing apical dead space. In case of reoperation in the future the above described technique, provides adequate surgical access and makes the second procedure less vigorous. It is well established that even the presence of a minimal apical dead space may lead to pneumothorax recurrence. Therefore, we prefer to apply mild negative suction on the intercostal drain and remove it only when the lung is fully expanded.

Since patients with SJMS may exhibit in the future recurrence of the pneumothorax as a result of formation and rupture of new bullae, or present with recurrent pulmonary infections requiring reoperation, we strongly suggest this surgical approach especially among younger individuals.

## Conclusion

SJMS can be associated with spontaneous pneumothorax and surgical therapy should be considered when there is no clinical or radiologic improvement of patient. Minimally invasive surgery with VAT bullectomy and apical pleurectomy should be regarded the treatment of choice; since SJMS is a continuous active inflammatory process and recurrence of pneumothorax from newly formed bullae may be observed, requiring reoperation.

## Consent section

Written informed consent was obtained from the patient for publication of this case report and accompanying images. No experimental research is reporting in this manuscript.

## Abbreviations

SJMS: Swyer-James-McLeod syndrome; VATS: Video-assisted thoracoscopic surgery; FEV1: Forced expiratory volume in 1 sec; FVC: Forced vital capacity; Tc: Technetium-99 m; BAL: Bronchoalveolar lavage; V/Q scan: Ventilation/perfusion scan; HCRT: High resolution computed tomography; LVRS: Lung volume reduction surgery.

## Competing interests

The authors declare that they have no competing interests.

## Authors’ contributions

All authors: 1) have made substantial contributions to conception and design, or acquisition of data, or analysis and interpretation of data; 2) have been involved in drafting the manuscript or revising it critically for important intellectual content; and 3) have given final approval of the version to be published.
